# The Ice Treatment

**Published:** 1890-03-01

**Authors:** 


### THE ICE TREATMENT.

With reference to the ice treatment of pneumonia noticed in the *Retrospect* of November 16th, we remarked as follows: "It must therefore be regarded as *sub judice*, even when viewed in the most favourable aspect." We observe that Dr. Hine, of Ealing, has written to a contemporary on the subject. He says granted the ice treatment may lower the temperature, does it not also depress the vital powers and diminish the *vis vitæ*? Dr. Hine can remember when venesection, tartar emetic, and mercurials were the orthodox remedies. Then came treatment by warmth, poultices, and stimulants, backed by nutriment, and he has no hesitation in declaring his faith in the last mode of treatment. And with reference to the application of ice to the chest in hœmoptysis, Dr. James More writes from Northamptonshire that he should be disposed to rely on full and frequent doses of ergot in all cases of hæmoptysis, in preference to any other mode of treatment.

